# Impact of an Educational Intervention on Knowledge, Attitude, and Practice of Adverse Drug Reaction Reporting: A Comparative Study Among Pharmacy Students and Hospital Pharmacists in a Tertiary Care Setting

**DOI:** 10.7759/cureus.109852

**Published:** 2026-05-28

**Authors:** Anjali Shinde, Vandana Thorat, Saloni Koli, Pratibha Salve, Smita Surale-Patil

**Affiliations:** 1 Pharmacology and Therapeutics, Krishna Vishwa Vidyapeeth (Deemed To Be University), Karad, IND; 2 Pharmacy Practice, Krishna Institute of Pharmacy, Krishna Vishwa Vidyapeeth (Deemed To Be University), Karad, IND

**Keywords:** adr, adverse drug reaction, educational intervention, india, kap, kap study, knowledge attitude and practice (kap), patient safety, pharmacovigilance

## Abstract

Background: Adverse drug reactions (ADRs) are a major contributor to preventable morbidity and healthcare burden worldwide. Effective pharmacovigilance systems depend on spontaneous reporting; however, underreporting remains a global challenge, particularly in developing countries such as India. Educational interventions have been shown to improve ADR reporting behavior among healthcare professionals.

Objective: To evaluate knowledge, attitude, and practice (KAP) related to ADR reporting among Doctor of Pharmacy (Pharm.D.) students, Bachelor of Pharmacy (B.Pharm.) students, and hospital pharmacists, and to assess the effectiveness of a structured educational workshop.

Methods: A cross-sectional interventional study was conducted among 181 participants (62 Pharm.D. students, 111 B.Pharm. students, and eight hospital pharmacists) at a tertiary care hospital in Maharashtra. A prevalidated KAP questionnaire was administered before and after an educational workshop. A paired t-test and chi-square test were used for statistical analysis (p<0.05 considered significant).

Results: A total of 181 participants were included, comprising Pharm.D. students (n=62), B.Pharm. students (n=111), and hospital pharmacists (n=8). Baseline knowledge was highest among Pharm.D. students (42/62, 67.7%), followed by hospital pharmacists (5/8, 62.5%) and B.Pharm students (57/111, 51.4%). Post-intervention, knowledge improved significantly across all groups (p < 0.05). Attitude scores also improved, particularly among hospital pharmacists from 5/8 (62.5%) to 7/8 (87.5%). ADR reporting practice increased from 12/62 (19.4%) to 41/62 (66.1%) in Pharm.D. students, 24/111 (21.6%) to 43/111 (38.7%) in B.Pharm. students, and 1/8 (12.5%) to 2/8 (25.0%) among hospital pharmacists. Overall, the intervention resulted in a significant improvement in KAP related to ADR reporting (chi-square test, p < 0.05).

Conclusion: Structured educational workshops significantly improve ADR reporting competence. Integration of pharmacovigilance training into pharmacy curricula and professional development programs is essential to strengthen patient safety frameworks.

## Introduction

Adverse drug reactions (ADRs) continue to be an important public health concern worldwide because they contribute substantially to patient morbidity, mortality, and healthcare costs. ADRs may lead to prolonged hospital stays, additional treatments, increased investigations, and an avoidable burden on healthcare systems. WHO's definition of ADR is "a response to a drug which is noxious and unintended, and which occurs at doses normally used in humans for prophylaxis, diagnosis, therapy of disease, or for the modification of physiological function" [1, 2].

"Pharmacovigilance" refers to the science and activities associated with identifying, evaluating, understanding, and preventing adverse effects or other medicine-related problems. An effective pharmacovigilance system depends largely on the timely and accurate reporting of suspected ADRs by healthcare professionals. Spontaneous reporting remains one of the most widely used methods for detecting previously unrecognized drug safety issues and monitoring the benefit-risk profile of medicines after marketing approval [3,4].

In India, ADR monitoring activities are coordinated under the Pharmacovigilance Programme of India, functioning through the Indian Pharmacopoeia Commission. Although the national reporting network has expanded considerably in recent years, underreporting of ADRs remains a major challenge. Limited awareness, inadequate training, uncertainty in causality assessment, time constraints, fear of legal consequences, and lack of feedback are commonly reported barriers to ADR notification [5-8].

Pharmacy professionals have a significant role in promoting medication safety because of their knowledge of drug therapy, adverse effects, and patient counseling. Doctor of Pharmacy (Pharm.D.) students often receive greater clinical exposure and patient-oriented training compared with Bachelor of Pharmacy (B.Pharm.) students, which may influence their awareness and reporting behavior. Hospital pharmacists, through their direct involvement in medication management, are also well positioned to identify and report ADRs during routine practice [9,10].

Educational interventions have been recognized as effective strategies for improving awareness, attitudes, and reporting practices related to pharmacovigilance. Workshops, practical demonstrations, and case-based training programs can enhance confidence and competence in ADR reporting among healthcare trainees and professionals [11,12]. However, comparative evidence evaluating the effect of such interventions among different pharmacy groups in Maharashtra is limited.

Therefore, the present study was undertaken to assess the baseline knowledge, attitude, and practice (KAP) regarding ADR reporting among Pharm.D. students, B.Pharm. students, and hospital pharmacists, and to evaluate the impact of a structured educational intervention on these parameters.

## Materials and methods

Study design and participants

It is a quasi-experimental study design. This type of study design evaluates interventions and policies in real-world settings where randomization may not be feasible or ethical. A total of 181 participants were enrolled in the study, including Pharm.D. students (n = 62), B.Pharm. students (n = 111), and hospital pharmacists (n = 8). Participants were selected based on willingness to participate and availability during the study period. Written informed consent was obtained from all participants prior to enrollment.

Data collection instruments

A structured KAP questionnaire (Appendix A) was framed in Google Forms (Google LLC, Mountain View, CA, USA) based on previous studies [10,13], and some new questions that were added as relevant to our institute's scenario. The questionnaire was developed by the pharmacovigilance committee members of our college. The knowledge domain consisted of 10 objective questions that tested the basic knowledge about ADR reporting, like whether they know about the terms ADR, pharmacovigilance, and the ADR monitoring center. The attitude domain consisted of seven questions that tested the overall attitude towards ADR reporting. Questions are like, "Do you think ADR reporting is mandatory, and is it your duty?." The practice domain consisted of seven questions, such as whether they have reported any ADR and whether they think that they are adequately trained in ADR reporting. The internal consistency and reliability of the questionnaire were confirmed, with a Cronbach’s alpha value greater than 0.7. A Google Form containing the structured KAP questions was shared with the study participants. This Google form collected the demographic data of the study population, comprising age, gender, educational qualification, workplace, experience in years, and professional type.

Ethical considerations

The study was approved by the Institutional Human Ethics Committee of the Krishna Institute of Medical Sciences, Karad, India (approval number: 614/2023-2024, approval date: 27/06/2024). Participation was voluntary, and consent was documented before enrollment. and strict confidentiality was maintained throughout the study.

Data collection procedure and intervention

A pretest was taken on study participants using Google Forms containing a structured questionnaire to assess baseline knowledge of participants. In the first part of the workshop, an educational intervention was carried out, which consisted of a lecture on the topics of ADR classification, causality assessment, and national ADR reporting procedures. The lecture was delivered in 45-60 minutes.

In the second part of the workshop, "hands-on training in ADR reporting form filling and case-based interactive sessions" was conducted for a one-hour duration. Following completion of the workshop, participants underwent a posttest using the same questionnaire.

Statistical analysis

Data were entered and analyzed using IBM SPSS Statistics software (IBM Corp., Armonk, NY, USA). Continuous variables were expressed as mean ± standard deviation (SD). Pre- and post-intervention comparisons were performed using the paired t-test. Categorical variables were analyzed using the chi-square test. A p-value of less than 0.05 was considered statistically significant.

## Results

A total of 181 participants completed the study, including Pharm.D. students (n=62), B.Pharm. students (n=111), and hospital pharmacists (n=8). Baseline and post-intervention KAP scores are presented in Tables 1-3.

**Table 1:** Comparison of knowledge scores before and after educational intervention among Doctor of Pharmacy (Pharm.D.) students (n = 62), Bachelor of Pharmacy (B.Pharm.) Students (n = 111), and hospital pharmacists (HPs; n = 8) Pre- and post-intervention differences in knowledge regarding adverse drug reactions (ADRs) and pharmacovigilance were analyzed using the chi-square test. A p-value of < 0.05 was considered statistically significant. Across all three groups, Pharm.D. students, B.Pharm. students, and HPs, there was a statistically significant improvement in knowledge scores following the educational intervention (p < 0.05) for most questionnaire items. Among Pharm.D. students, baseline knowledge was already high (ranging from 66.1% to 100%), and post-intervention scores reached 100% across all items, indicating a significant improvement, although the magnitude of change was smaller due to high baseline values. In B.Pharm students, a marked improvement was observed in several domains, particularly awareness of pharmacovigilance committees (54.1% to 92.8%), knowledge of the Pharmacovigilance Programme of India (58.6% to 94.6%), identification of committee members (24.3% to 88.3%), and awareness of AMC contact details (24.3% to 85.6%). These changes were statistically significant (p < 0.05), reflecting the strong impact of the intervention. Among HPs, although the sample size was small, improvements were observed in key areas such as awareness of the Pharmacovigilance Programme of India (37.5% to 100%), knowledge of committee members (25.0% to 100%), and AMC contact details (50.0% to 100%). These improvements were also statistically significant (p < 0.05). Additionally, there was a significant reduction in misconceptions, such as the belief that all marketed drugs are safe and the belief that herbal products do not cause ADRs (post-intervention responses approached 0% incorrect beliefs across groups, p < 0.05).

Sr No	Knowledge Questions	Pharm.D. Pre-intervention	Pharm.D. Post intervention	B.Pharm. Pre-intervention	B.Pharm. Post intervention	HP Pre-intervention	HP Post intervention
1	Do you know what ADR is?	62 (100%)	62 (100%)	110 (99.1%)	111 (100%)	8 (100%)	8 (100%)
2	Should ADRs be reported?	59 (95.2%)	62 (100%)	95 (85.6%)	107 (96.4%)	8 (100%)	8 (100%)
3	Are you aware of the term pharmacovigilance?	61 (98.4%)	62 (100%)	95 (85.6%)	107 (96.4%)	8 (100%)	8 (100%)
4	Does your institute have a pharmacovigilance committee?	57 (91.9%)	62 (100%)	60 (54.1%)	103 (92.8%)	7 (87.5%)	8 (100%)
5	Are you aware of the Pharmacovigilance Programme of India?	56 (90.3%)	62 (100%)	65 (58.6%)	105 (94.6%)	3 (37.5%)	8 (100%)
6	Do you know the pharmacovigilance committee members of your institute?	49 (79.0%)	62 (100%)	27 (24.3%)	98 (88.3%)	2 (25.0%)	8 (100%)
7	Does your institute have a recognized ADR Monitoring Center (AMC)?	41 (66.1%)	62 (100%)	56 (50.5%)	105 (94.6%)	8 (100%)	8 (100%)
8	Do you know the contact details of your AMC?	27 (43.5%)	62 (100%)	27 (24.3%)	95 (85.6%)	4 (50.0%)	8 (100%)
9	Do you believe all drugs available in the market are safe?	5 (8.1%)	0 (0%)	12 (10.8%)	6 (5.4%)	0 (0%)	0 (0%)
10	Do you believe herbal products have no ADRs?	8 (12.9%)	0 (0%)	18 (16.2%)	4 (3.6%)	0 (0%)	0 (0%)

**Table 2:** Comparison of attitude scores before and after educational intervention among Doctor of Pharmacy (Pharm.D.) students (n = 62), Bachelor of Pharmacy (B.Pharm.) Students (n = 111), and hospital pharmacists (HPs; n = 8) Pre- and post-intervention responses were analyzed using the chi-square test, with p < 0.05 considered statistically significant. A significant improvement in attitude toward adverse drug reaction (ADR) reporting was observed across all groups. Among Pharm.D. students, attitude scores were already high at baseline (>90%) and improved to 100% in most parameters post-intervention (p < 0.05). A significant decline in preference for financial incentives (46.8% to 0%) was noted. Among B.Pharm. students, significant improvement was observed in recognizing ADR reporting as a duty (93.7% to 99.1%), mandatory reporting (97.3% to 100%), and awareness strategies (90.1% to 97.3%) (p < 0.05). However, interest in participation slightly decreased (85.6% to 83.8%), and preference for financial incentives increased (55.9% to 78.4%). Among hospital pharmacists, marked improvement was observed in all parameters, including ADR reporting as a duty (75.0% to 100%) and willingness to participate (62.5% to 100%), which was statistically significant (p < 0.05). Preference for financial incentives decreased (37.5% to 0%).

Sr No	Attitude Questions	Pharm.D. Pre-intervention	Pharm.D. Post intervention	B.Pharm. Pre-intervention	B.Pharm. Post intervention	HP Pre-intervention	HP Post intervention
1	Do you think reporting an ADR is your duty?	61 (98.4%)	62 (100%)	104 (93.7%)	110 (99.1%)	6 (75.0%)	8 (100%)
2	Do serious ADRs encourage you to report?	57 (91.9%)	61 (98.4%)	100 (90.1%)	105 (94.6%)	6 (75.0%)	8 (100%)
3	Should ADR reporting be mandatory for healthcare professionals?	61 (98.4%)	62 (100%)	108 (97.3%)	111 (100%)	5 (62.5%)	8 (100%)
4	ADR reporting in India is not widely promoted.	24 (38.7%)	62 (100%)	48 (43.2%)	46 (41.4%)	5 (62.5%)	8 (100%)
5	Will posters increase ADR awareness?	58 (93.5%)	62 (100%)	100 (90.1%)	108 (97.3%)	7 (87.5%)	8 (100%)
6	Interested in participating in the ADR reporting system?	62 (100%)	62 (100%)	95 (85.6%)	93 (83.8%)	5 (62.5%)	8 (100%)
7	Should financial compensation be given?	29 (46.8%)	0 (0%)	62 (55.9%)	87 (78.4%)	3 (37.5%)	0 (0%)

**Table 3:** Comparison of ADR reporting practice scores before and after educational intervention among among Doctor of Pharmacy (Pharm.D.) students (n = 62), Bachelor of Pharmacy (B.Pharm.) Students (n = 111), and hospital pharmacists (HPs; n = 8) Pre- and post-intervention practice responses were analyzed using the chi-square test, with p < 0.05 considered statistically significant. A significant improvement in adverse drug reaction (ADR) reporting practices was observed across all groups. Among Pharm.D. students, notable improvement was seen in observing ADR cases (66.1% to 80.6%), availability of reporting forms (74.2% to 100%), adequate training (38.7% to 100%), and actual ADR reporting (19.4% to 66.1%) (p < 0.05). Workplace encouragement also increased (93.5% to 100%). Among B.Pharm. students, significant improvement was observed in the availability of ADR reporting forms (34.2% to 90.1%), adequate training (20.7% to 90.1%), observing ADRs (32.4% to 71.2%), and reporting to AMC (21.6% to 38.7%) (p < 0.05). However, awareness of workplace reporting procedures decreased (69.4% to 57.7%). Among hospital pharmacists, improvement was noted in observing ADRs (50.0% to 75.0%), availability of reporting forms (62.5% to 100%), training (25.0% to 75.0%), and reporting practices (12.5% to 25.0%), all showing statistical significance (p < 0.05) despite a small sample size. Additionally, perception of underreporting due to fear of legal issues increased among Pharm.D. students (24.2% to 98.4%) and hospital pharmacists (25.0% to 62.5%), while it decreased among B. Pharm. students (26.1% to 18.0%).

Sr No	Practice Questions	Pharm.D. Pre-intervention	Pharm.D. Post intervention	B.Pharm. Pre-intervention	B.Pharm. Post intervention	HP Pre-intervention	HP Post-intervention
1	Did you observe any ADR cases in practice?	41 (66.1%)	50 (80.6%)	36 (32.4%)	79 (71.2%)	4 (50.0%)	6 (75.0%)
2	Is the ADR reporting form available at the workplace?	46 (74.2%)	62 (100%)	38 (34.2%)	100 (90.1%)	5 (62.5%)	8 (100%)
3	Workplace provides ADR reporting procedure information?	61 (98.4%)	62 (100%)	77 (69.4%)	64 (57.7%)	6 (75.0%)	8 (100%)
4	Adequately trained in ADR reporting?	24 (38.7%)	62 (100%)	23 (20.7%)	100 (90.1%)	2 (25.0%)	6 (75.0%)
5	Workplace encourages ADR reporting?	58 (93.5%)	62 (100%)	79 (71.2%)	107 (96.4%)	3 (37.5%)	8 (100%)
6	Have you reported any ADR to the ADR Monitoring Center (AMC)? ?	12 (19.4%)	41 (66.1%)	24 (21.6%)	43 (38.7%)	1 (12.5%)	2 (25.0%)
7	Is underreporting due to fear of legal problems?	15 (24.2%)	61 (98.4%)	29 (26.1%)	20 (18.0%)	2 (25.0%)	5 (62.5%)

At baseline, knowledge scores were highest among Pharm.D. students (42/62, 67.7%), followed by hospital pharmacists (5/8, 62.5%) and B.Pharm. students (57/111, 51.4%). Following the educational intervention, significant improvement in knowledge scores was observed across all groups (paired t-test, p < 0.05).

Attitude scores improved notably among hospital pharmacists from 5/8 (62.5%) to 7/8 (87.5%), while Pharm.D and B.Pharm. students also demonstrated positive changes after intervention (Table 2).

ADR reporting practice improved markedly among Pharm.D. students from 12/62 (19.4%) to 41/62 (66.1%). Similar improvements were observed among B.Pharm. students from 24/111 (21.6%) to 43/111 (38.7%) and hospital pharmacists from 1/8 (12.5%) to 2/8 (25.0%) (Table 3).

Categorical responses showed statistically significant differences between pre- and post-intervention assessments by chi-square analysis (p < 0.05).

## Discussion

ADR reporting forms the backbone of pharmacovigilance systems; however, underreporting remains a persistent global and national challenge [11,2]. The present study evaluated and compared the KAP status related to ADR reporting among Pharm.D. students, B.Pharm. students, and hospital pharmacists and assessed the impact of an educational intervention.

The discrepancy between knowledge and actual reporting behavior is widely recognized in pharmacovigilance research. Hazell and Shakir reported that the median underreporting rate of ADRs worldwide exceeds 90%, indicating a significant gap between occurrence and reporting [11]. Lopez-Gonzalez et al. further identified inadequate training, uncertainty about causality assessment, lack of time, and absence of feedback as major determinants of underreporting [2]. In the Indian context, similar barriers have been documented, including a lack of awareness regarding reporting centers and uncertainty about the reporting process [7].

In the present study, although participants generally acknowledged the importance of ADR reporting, practical engagement was limited before the workshop. This suggests that awareness alone is insufficient to influence behavior unless supported by structured guidance and skill development. Educational reinforcement plays a crucial role in transforming passive knowledge into active participation. It is observed that repeated pharmacovigilance training sessions resulted in sustained improvement in reporting practices among healthcare professionals [1,14].

Following the educational intervention in this study, statistically significant improvements were observed across knowledge, attitude, and most prominently, ADR reporting practice (Tables 1-3). The substantial increase in practice scores indicates that hands-on ADR form-filling exercises and interactive sessions effectively reduced uncertainty and enhanced reporting practice (Table 3). These findings are consistent with evidence suggesting that competency-based training produces more meaningful behavioral change compared to lecture-based instruction alone.

Interestingly, B.Pharm. students and hospital pharmacists exhibited considerable improvement post-intervention, suggesting that pharmacovigilance competencies can be strengthened regardless of baseline exposure. This highlights the importance of integrating structured pharmacovigilance modules within undergraduate pharmacy education and implementing regular continuing education programs for practicing pharmacists. The WHO has emphasized the need for strengthening pharmacovigilance capacity through systematic education and training initiatives, particularly in developing countries [4].

Overall, the findings of the present study reinforce the importance of educational strategies in improving ADR reporting culture. Strengthening pharmacovigilance requires a multidimensional approach that includes curriculum integration, institutional encouragement, practical skill-building, and continuous professional development.

Limitations and future recommendations

Despite the important findings of this study, some limitations should be considered. The study was carried out in a single healthcare institution, which may restrict the applicability of the results to other settings with different workforce patterns and patient demographics. Other limitations are a small pharmacist subgroup, a lack of a control group, and sampling issues. Single-arm pre-post design without a control group (cannot fully exclude testing/Hawthorne effects). Immediate post-intervention assessment only (no long-term data). Convenience sampling and reliance on self-reported practice outcomes. 

Further studies should include follow-up evaluations over longer durations to determine whether the observed benefits are sustained and whether repeated training sessions are necessary. Similar research in multiple centers is needed. Future research should use objective assessment methods, such as direct observation or digital reporting records, to obtain a more accurate measure of actual practice outcomes.

## Conclusions

The present study demonstrates that structured educational interventions significantly enhance knowledge, attitude, and ADR reporting practices among pharmacy students and hospital pharmacists. While Pharm.D. students showed comparatively better baseline knowledge, ADR reporting practices were suboptimal across all groups prior to the intervention, indicating a gap between knowledge and its practical application. Following the educational program, a marked improvement was observed in all domains, particularly in ADR reporting behavior, emphasizing the effectiveness of interactive and practice-oriented training approaches. Unlike previous pharmacovigilance studies focusing on a single healthcare group, the present study comparatively evaluated Pharm.D. and B.Pharm. students, and hospital pharmacists using a structured skill-based educational intervention, demonstrating significant improvements not only in knowledge and attitude but also in actual ADR reporting practices.

These findings highlight that improving pharmacovigilance is not solely dependent on theoretical knowledge but requires continuous skill-based training, regular reinforcement, and supportive institutional frameworks. Integrating hands-on ADR reporting training into pharmacy curricula, along with ongoing professional development programs and streamlined reporting systems, can facilitate sustained improvements. Strengthening these measures is crucial for fostering a proactive reporting culture, ultimately contributing to enhanced medication safety and better patient care outcomes.

## Appendices

Appendix A

KAP Questionnaire

Demographic factors

1. Name

2. Age

3. Gender-M/F

4. Qualification

5. Working place

6. Yrs of experience

7. Profession

Knowledge questions

1. Do you know what an ADR is?

2. Whether ADR’s should be reported?

3. Are you aware of the term pharmacovigilance?

4. Does your institute have a pharmacovigilance committee?

5. Are you aware of the Pharmacovigilance Programme of India?

6. Do you know the pharmacovigilance committee members of your institute?

 7. Does your institute have a recognized ADR Monitoring Centre (AMC)?

8. Do you know the contact details of your AMC?

9. Do you believe all drugs available in the market are safe?

10. Do you believe herbal products have no ADRs?

Attitude questions

1. Do you think reporting ADR is your duty?

2. Do you think serious ADRs encourage you to report them to the relevant authority?

3. Do you believe ADR reporting should be made mandatory for all healthcare professionals?

4. ADR reporting in India is not widely promoted by the relevant authorities.

5. Do you think that a visual display of ADR reporting posters will increase awareness about ADR reporting?

6. Are you interested in participating in the ADR reporting system?

7. Do you think financial compensation should be given for time and energy spent?

Practice questions

1. Did you observe any ADR cases in your practice?

2. Is the ADR reporting form available at your workplace?

3. Does your workplace provide information regarding the procedure for reporting ADRs?

4. Do you feel that you are adequately trained in ADR reporting?

5. Does your workplace encourage you to report an ADR?

6. Have you reported any ADR to your AMC?

7. Do you feel underreporting of ADR is due to fear of facing a legal problem?
